# Mitochondrial apolipoprotein A-I binding protein alleviates atherosclerosis by regulating mitophagy and macrophage polarization

**DOI:** 10.1186/s12964-022-00858-8

**Published:** 2022-05-07

**Authors:** Meng Duan, Hainan Chen, Linjie Yin, Xiao Zhu, Petr Novák, Yuncheng Lv, Guojun Zhao, Kai Yin

**Affiliations:** 1 Department of Cardiology, The Second Affiliated Hospital of Guilin Medical University, Guangxi Key Laboratory of Diabetic Systems Medicine, Guilin, 541100 Guangxi China; 2 Guangxi Key Laboratory of Diabetic Systems Medicine, Guilin Medical University, Guilin, Guangxi China; 3 Research Lab of Translational Medicine, Hengyang Medical School, University of South China, Hengyang, China; 4 Center for Stem Cell Biology and Tissue Engineering, Key Laboratory for Stem Cells and Tissue Engineering, Ministry of Education, Sun Yat-Sen University, Guangzhou, China; 5 The Sixth Affiliated Hospital of Guangzhou Medical University, Qingyuan City People’s Hospital, Qingyuan, 511518 Guangdong China

**Keywords:** AIBP, Mitophagy, Atherosclerosis, Macrophage polarization

## Abstract

Apolipoprotein A-I binding protein (AIBP), a secreted protein, has been shown to play a pivotal role in the development of atherosclerosis. The function of intracellular AIBP, however, is not yet well characterized. Here, we found that AIBP is abundantly expressed within human and mouse atherosclerotic lesions and exhibits a distinct localization in the inner membrane of mitochondria in macrophages. Bone marrow-specific AIBP deficiency promotes the progression of atherosclerosis and increases macrophage infiltration and inflammation in low-density lipoprotein receptor-deficient (LDLR^−/−^) mice. Specifically, the lack of mitochondrial AIBP leads to mitochondrial metabolic disorders, thereby reducing the formation of mitophagy by promoting the cleavage of PTEN-induced putative kinase 1 (PINK1). With the reduction in mitochondrial autophagy, macrophages polarize to the M1 proinflammatory phenotype, which further promotes the development of atherosclerosis. Based on these results, mitochondrial AIBP in macrophages performs an antiatherosclerotic role by regulating of PINK1-dependent mitophagy and M1/M2 polarization.

**Video Abstract**

**Supplementary Information:**

The online version contains supplementary material available at 10.1186/s12964-022-00858-8.

## Background

Atherosclerosis, the main cause of cardiovascular disease (CVD), is regarded as a chronic inflammatory process driven by lipid accumulation in the arterial wall [1, 2]. Macrophages, which are the major inflammatory cells, not only play a crucial role in lipid metabolism but are also implicated in triggering inflammation [3]. Macrophage polarization is a multifactorial process in which a large number of factors are involved to produce different activation scenarios [4–6]. Macrophages have long been divided into proinflammatory M1 macrophages and anti-inflammatory M2 macrophages [6]. Currently, the functional polarization of macrophages into only two groups is an oversimplified description of macrophage heterogeneity and plasticity. However, with the advent of single-cell sequencing technology, the type of macrophages is no longer limited to the M1/M2 classification, and the heterogeneity of macrophages in disease progression can be detected more accurately [7]. Despite numerous challenges, the reversibility of polarization still has critical therapeutic value, especially in atherosclerosis, in which the M1/M2 imbalance plays a pathogenic role.

Apolipoprotein A-I binding protein (AIBP) was discovered in a screen of proteins closely associated with apolipoprotein A-I (apoA-I) [8]. Previous studies by our group and others have shown that extracellular AIBP promotes cholesterol efflux from foam cells by inducing the binding of ATP-binding cassette transporter A1 (ABCA1) to apoA-I or inhibiting ABCA1 degradation [9–12]. Extracellular AIBP exerts an anti-inflammatory effect by inhibiting Toll-like receptor 4 (TLR4)-mediated proinflammatory signaling [13–15]. Interestingly, AIBP also exists in mitochondria and plays a vital role in metabolic repair [16, 17]. In addition, a study found that AIBP gene mutation results in a serious defect in mitochondrial nicotinamide adenine dinucleotide phosphate (NAD (P) HX), which is reduced by the hydrate repair system and abnormal metabolism [18]. However, the exact function of mitochondrial AIBP and its precise role in atherosclerosis remain unclear.

In this study, we generated bone marrow-specific AIBP/LDLR double-deficient (AIBP^ΔBMK^/LDLR^−/−^) mice and found that AIBP deletion increased the number of M1 proinflammatory macrophages and atherosclerotic lesion size. In bone marrow-derived macrophages (BMDMs), we found that AIBP showed obvious colocalization with mitochondrial proteins. Furthermore, the lack of mitochondrial AIBP in macrophages caused mitochondrial metabolism disorders, leading to increased PINK1 protein cleavage and the inhibition of mitophagy. The lack of mitochondrial AIBP eventually led to the conversion of macrophages to the M1 type, which exacerbated inflammation. Overall, these dates suggest that mitochondrial AIBP may be a novel therapeutic target for the prevention of atherosclerosis.

## Materials and methods

### Animals

AIBP knockout (AIBP^−/−^) mice on a C57BL6/N background were established by crossing AIBP^+/−^ mice purchased from Cyagen (China). Briefly, AIBP is located on chromosome 3 of mice. CRISPR/Cas9 technology was used to design sgRNAs, and AIBP^+/−^ mice were obtained through the application of high-throughput electrotransfer fertilization. LDLR^−/−^ mice (C57BL/6 J background) were obtained from the Model Animal Research Center of Nanjing University. The mice were housed in the Animal Facility of University of South China (4 per standard cage at room temperature and maintained on a 12:12 h light:dark cycle, with lights on at 07:00). Both food and water were available ad libitum. All protocols described in this study were approved by the Institutional Animal Care and Use Committee of University of South China. All animal experiments were conformed to the international guidelines for the ethical use of laboratory animals.

### Human tissue samples

Human tissue samples were obtained from human coronary arteries with a protocol approved by the First Affiliated Hospital of University of South China.

### Bone marrow transplantation

At 8 weeks of age, male LDLR^−/−^ mice were irradiated with 2 doses of 6.5 Gy from a cesium g source with a 4 h time interval [19]. At 24 h after irradiation, the mice were injected via a tail vein with 5–10 × 10^6^ bone marrow (BM) cells in DMEM (Dulbecco's modified Eagle's medium, Cat#11965092, Gibco) containing 2% FBS (Cat#10099, Gibco) from control (C57BL/6 N background) and AIBP^−/−^ mice via the tail vein. Seven days before irradiation and 2 weeks after irradiation, a sulfamethoxazole and trimethoprim oral suspension (200 mg/ml/40 mg/ml; Cat# A2487, Sigma) to acidified water (pH 2.4–3.1) and provided at a dose of 1 mg/0.2 mg per 25 g mouse [20]. The mice were allowed to recover for 4 weeks after BM transplantation before feeding them a high-fat diet (HFD, Cat#TD88137, Harlan Teklad) 8 weeks or the indicated times to promote atherosclerosis. After the recovery period, peripheral blood was collected, and DNA was extracted to determine the efficiency of BM reconstitution by quantifying wild-type AIBP DNA compared with actin (for the AIBP^−/−^ and control BM transplantation). For all BM transplantation studies, reconstitution was > 95%. For all studies, animals were randomized according to their genotype or treatment.

### Atherosclerosis analysis

The assessment was performed as described previously [21]. After receiving Ethics Committee approval, human atherosclerotic plaques were obtained following carotid endarterectomy. The mouse hearts were dissected and embedded in optimal cutting temperature compound (Sakura Tissue-Tek, U.S.A), and 5-μm-thick sections of aortic roots were produced with a Leica CM3050 S Research Cryostat (Leica, Germany) for Oil Red O and HE staining. The lesion areas on the aorta and aortic roots were quantified using ImageJ 2X software (Media Cybernetics, Bethesda, MD).

### Specific steps for the isolation, culture and polarizationof BMDMs

1. L-929 cell conditioned medium [22]was obtained by inoculating 5 × 10^5^ L-929 cells (Chinese Academy of Sciences, Shanghai, China) in a cell culture flask and incubating them with complete medium consisting of DMEM, 10% FBS and 1% penicillin/streptomycin (Cat#15140122, Gibco) at 37 °C in a 5% CO2 cell incubator for 8 days without changing the medium. Next, the cell supernatant was collected with a 0.2 µm syringe filter and frozen.
2. Separation of bone marrow (BM) cells [23]: The mice were euthanized by cervical dislocation, and the thighs were cut circularly with ophthalmic scissors. The muscles on the legs were bluntly separated, and the femur and tibia were removed. The femur and tibia were separated in a Petri dish containing PBS, and the muscle and fascia were removed. The bones were washed with PBS and immersed in complete medium on ice. The end of the bone was cut with scissors, and a syringe fitted with a 25-gauge needle and filled with 5 mL of complete medium was used to flush the contents of the medullary cavity into a fresh petri dish. The rinse solution was filtered through a 75 µm stainless steel screen to obtain a single-cell suspension.
3. Induction and formation of BMDMs [24]: First, a special medium was prepared for BMDMs by adding 15% L-929 cell supernatant, 10% FBS, and 1% penicillin/streptomycin mixture to DMEM. The special medium was used to culture BMDMs. Bone marrow cells were resuspended, counted, and seeded in a six-well plate at a density of 2–5 × 10^6^ cells/ml. Cells were cultured in a 37 °C, 5% CO2 cell incubator, and the media were replaced with fresh media every 3 days. The special medium for BMDMs was used to observe the changes of cell morphology under a light microscope daily. Generally, the cells differentiated into macrophages by the seventh day of culture. At this time, the cells exhibited obvious pseudopodia.
4. Macrophage polarization [25]: Macrophages were stimulated with lipopolysaccharide (LPS; 50 ng/mL; Cat# No. L2630, Sigma) and IFNγ (20 ng/ml; Cat# 300-02, PeproTech) for 6 h, and IL-4 (10 ng/mL; Cat# No. 214-14, PeproTech) for 16 h.

### Mouse peritoneal macrophages (MPMs) extraction

Mouse peritoneal macrophages (MPMs) were collected from AIBP^ΔBMK^/LDLR^−/−^ and AIBP^WT^/LDLR^−/−^ mice via peritoneal lavage 4 days after the intraperitoneal injection of 4% thioglycolate (1 ml, BD Biosciences, Franklin Lakes, NJ, USA). Five minutes later, the mice were euthanized, and the peritoneal fluid was removed and centrifuged at 250*g* for 8 min at room temperature. The cell pellet was resuspended in RPMI-1640 (Cat# C11875500BT, Gibco) supplemented with 10% FBS and incubated in six-well plates at 37 °C in an atmosphere of 5% CO_2_ for 2 h. Adherent cells were cultured and used in subsequent experiments.

### Lentiviral plasmid construction and lentiviral infection

The empty lentivirus vectors (Null), lentivirus carrying full-length AIBP, lentivirus carrying the mitochondrial AIBP deletion sequence (AIBP^ΔMLS^) vectors and lentivirus carrying full-length PINK1 were packaged by Genechem [26]. The overexpression constructs were cloned into the pCDH-CMV-MCS-EF1-Puro vectors. HEK293T cells were transiently transfected with constructs (pCDH-CMV-MCS-EF1-Puro), psPAX2 and pMD2.G to obtain lentiviral particles. BMDMs were infected with lentiviruses to overexpress target genes of interest. All protein overexpression sequences are listed in Additional file 2: Table S1.

### Subcellular fractionation

Mitochondria were isolated by differential centrifugation [27]. Protease treatment was performed on the intact mitochondrial fraction isolated from BMDMs by digestion with 100 ng/ml proteinase K (Cat# ST532, Beyotime) in the presence or absence of 1% Triton X-100 (Cat# ST797, Beyotime) on ice followed by Western blot analysis. Alkaline extraction was performed using Na_2_CO_3_ to separate OM and IM vesicles from BMDM mitochondria.

### Flow cytometry

Flow cytometry was conducted as described previously [28]. Blood samples were collected from the tail vein into EDTA-coated tubes and immediately placed on ice. For the analysis of blood leukocyte subsets, tubes were incubated at 4 °C throughout the procedure, unless indicated otherwise. Red blood cells (RBCs) were lysed (BD Pharm Lyse, Cat# 55899, BD Bioscience), and white blood cells were centrifuged, washed, and resuspended in HBSS (Cat# C0218, Beyotime). The cells were stained with a cocktail of antibodies against CD11b (M1/70, Cat^#^101205, BioLegend) and F4/80 (Cat^#^T45-2342, BD Biosciences). For identification of the macrophage phenotype, macrophages were extracted from the plaques and isolated as previously described [29, 30]. The macrophages were stained with a cocktail of antibodies against CD206 (Cat^#^C068C2, BioLegend) and CD86 (Cat^#^105005, BioLegend). Data were acquired with a FACSCantoII (Becton Dickinson) and analyzed using FlowJo software (Tree Star).

### Immunofluorescence staining

For immunofluorescence staining, tissue sections were fixed with 4% paraformaldehyde for 20 min, incubated with permeabilization solution (0.1% Triton × 100 and 0.1% sodium citrate) on ice for 2 min and blocked with horse serum for 2 h. Sections were stained with antibodies against Mac3 (1:200, Cat^#^ ab199947, Abcam), CD31 (1:200, Cat^#^ sc-376764, Santa Cruz Biotechnology), α-SMA (1:200, Cat^#^sc-53142, Santa Cruz Biotechnology), or AIBP (1:200, Cat^#^ ab75114, Abcam) followed by detection with fluorochrome-conjugated secondary antibodies (fluorescein (FITC)–conjugated Affinipure goat anti-rabbit IgG (H + L), Cat^#^ SA00003-2, Proteintech; fluorescein (FITC)–conjugated Affinipure goat anti-mouse IgG (H + L), Cat^#^ SA00003-1, Proteintech; Cy3–conjugated Affinipure goat anti-rabbit IgG (H + L), Cat^#^ SA00009-2, Proteintech; and Cy3–conjugated Affinipure goat anti-mouse IgG (H + L), Cat^#^ SA00009-1, Proteintech). Sections were counterstained with DAPI (Cat# BS097, Biosharp), coverslipped and then scanned with a fluorescence microscope (IX70; Olympus, Tokyo). Negative controls were obtained by incubating tissue sections with the corresponding secondary antibodies alone. Images were analyzed using ImageJ 2X software (Media Cybernetics, Bethesda, MD).

### *ΔΨm*

To determine the changes in mitochondrial membrane potential, JC-1 dye (Cat^#^ C2006, Beyotime) was used to determine changes in the mitochondrial membrane potential. Cells were collected and washed with PBS two times. Cells were incubated with the JC-1 working stock solution for 20 min at 37 °C and then washed with JC-1 buffer solution twice. Cells stained with JC-1 were detected using flow cytometry or fluorescence microscopy.

### Measurement of ROS levels

Intracellular ROS levels were observed with the fluorescent dye 2′,7′-dichlorodihydrofluorescein diacetate (DCFH-DA, Cat^#^ S0033M, Beyotime). Briefly, the treated cells were incubated with DMEM containing 10 µM DCFH-DA for 30 min at 37 °C. After washing, the labeled cells were observed using a fluorescence microscope (IX70; Olympus, Tokyo) or a flow cytometer to detect the ROS levels.

### Assessment of ATP concentration

After stimulation and/or treatment with the appropriate reagents, the ATP concentration in cells was measured using an ATP Assay Kit (Cat# S0026B, Beyotime) [31]. A total of 3 × 10^4^ cells were cultivated in a 24-well plate for 24 h. Then, the cells were collected, mixed with cell lysis buffer, incubated for 10 min and centrifuged at 12000 g at 4 °C for 5 min. The ATP concentration in the supernatant was tested by incubating the sample with 100 μL of the solution provided in the kit and recorded using a luminometer (Maxwell Technologies Inc., CA, USA).

### ELISA analysis of cytokine levels

Mouse plasma was collected and stored at − 20 °C until analysis. Plasma concentrations of IL-1β, IL-6, IFN-α, IL-18, IL-12, IL-10 and IL-4 were measured using enzyme-linked immunosorbent assays (ELISAs) (DuoSet ELISA Development System, R&D Systems) according to the manufacturer’s instructions. The cytokine standards were used to generate standard curves. Quantitative determinations were performed in one experiment.

### Western blotting assay

The tissues were ground in liquid nitrogen. The ground tissues and cells were lysed in RIPA buffer (Cat# P0013B, Beyotime) containing 0.1 mmol/L PMSF (Cat# P1005, Beyotime) on ice for 30 min. After centrifugation at 12,000 rpm and 4 °C for 10 min, the supernatant was collected, and protein concentrations in the lysates were measured with the BCA assay (Cat# P0012S, Beyotime). The same amounts of total proteins were subjected to SDS–PAGE, followed by electroblotting onto polyvinylidene difluoride membranes (Millipore Corporation, USA.). The membranes were incubated with antibodies against AIBP (1:1000, Cat^#^ ab75114, Abcam), LC3b (1:1000, Cat^#^ ab63817, Abcam), p62 (1:1000, Cat^#^ 109012, Abcam), TOM20 (1:1000, Cat^#^ 186735, Abcam), PINK1 (1:1000, Cat^#^ ab23707, Abcam), Parkin (1:1000, Cat^#^ 77924, Abcam), β-actin (1:5000, Cat^#^ 66009-1-Ig, Proteintech), COXIV (1:5000, Cat^#^ 11242-1-AP, Proteintech), Lamin-B (1:500, Cat^#^ ab32535, Abcam), SMAC (1:1000, Cat^#^ 15108S, Cell Signaling Technology) and VDAC (1:1000, Cat^#^ 4661S, Cell Signaling Technology). After washes with PBS, the membranes were incubated with the appropriate secondary antibodies: HRP-labeled goat anti-mouse IgG (H + L) (1:2000, Cat# A0258, Beyotime) or HRP-labeled rabbit anti-mouse IgG (H + L) (1:1000, Cat# A0208, Beyotime). The signals were visualized with a chemiluminescence method using BeyoECL Star (Cat# P0018AM, Beyotime). β-Actin and TOM20 served as internal controls.

### RNA preparation and real-time PCR

Total RNA was extracted from BMDMs or primary peritoneal macrophages using TRIzol reagent (Cat# R0016, Beyotime). Reverse transcription was performed using 1 μg of total RNA with the PrimeScript RT reagent kit and gDNA eraser (Cat# RR047A, TaKaRa). Quantitative PCR (qPCR) was performed in triplicate to amplify specific genes and normalized to the β-actin level. Each PCR contained 0.8 μl (10 μM) of forward and reverse primers and 10 μl of 2 × SYBR Green PCR Master Mix for a final volume of 20 μl. PCR was performed with a Thermal Cycler Dice Real Time System (TP-800, TaKaRa, China). The generation of specific PCR products was confirmed by conducting a melting curve analysis. Relative gene expression was measured using the comparative Ct method, X = 2^−ΔΔCt^. Primer sequences are shown in Additional file 3: Table S2.

### Respiration and glycolysis

For the real-time analysis of the ECAR and OCR, BMDMs were analyzed with an XF-24 Extracellular Flux Analyzer according to the manufacturer’s instructions (Seahorse Bioscience). Briefly, cells were seeded at a density of 60,000 cells/well 24 h before the assay. For OCR measurements, media were changed to XF base medium (Seahorse Bioscience) containing 25 mM glucose (Sigma), 2 mM L-glutamine and 1 mM sodium pyruvate (Invitrogen) 1 h before the analysis. Five consecutive measurements were obtained under basal conditions. Mitochondrial respiration was further characterized after the sequential addition of 1 μg/mL oligomycin A (Santa Cruz Biotechnologies) to inhibit mitochondrial ATP synthase, 3 μM FCCP (fluoro-carbonyl cyanide phenylhydrazone) (Santa Cruz Biotechnologies), a protonophore that uncouples ATP synthesis from oxygen consumption by the electron-transport chain, and 100 nM rotenone (Sigma–Aldrich), which inhibits the electron transport chain. Three consecutive measurements were recorded after each sequential treatment. The instrumental background was measured in separate control wells under the same conditions without biological material. Metabolic rates were normalized to cell numbers.

Similarly, for the analysis of glycolysis characteristics, media were changed to XF base medium (Seahorse Bioscience) containing 2 mM L-glutamine (Invitrogen) 1 h prior to the analysis. The ECAR was measured after the sequential addition of 10 mM glucose to induce glycolysis, 1 μg/mL oligomycin to force cells to increase glycolysis to the maximal rate (glycolytic capacity), and 100 mM 2-DG, a glycolysis inhibitor.

### Coimmunoprecipitation (coIP)

For the AIBP/AIBP^(ΔMLS)^ and TIM23 coIP, 1–2 × 10^6^ 293 T cells were plated in 6-cm dishes and transfected with 3 μL of Lipofectamine 2000 (Cat# 11668019, Invitrogen) according to the manufacturer’s protocol. The AIBP/TIM23 coIP was performed using cells transfected with 0.25 μg of AIBP-FLAG/AIBP^(ΔMLS)^-Flag, 1 μg of PINK1-HA [16], and the corresponding empty vector controls. Twenty-four hours after transfection, the cells were washed twice with PBS, lysed in 500 μL of coIP buffer (150 mM NaCl, 25 mM HEPES, 0.2% NP40, and 10% glycerol) containing 1 mM DTT and protease inhibitors (1 mM PMSF, 1 μg/mL aprotinin, 10 μg/mL pepstatin and 1 μg/mL leupeptin), incubated for 20 min on ice and clarified by centrifugation at 15,000 rpm for 15 min at 4 °C. A sample was aliquoted as the input, and the remaining lysate was incubated for 6 h at 4 °C with 0.5 μg of anti-FLAG antibody (Cat# F7425, Sigma–Aldrich) or anti-HA antibody (Cat# 2367, Cell Signaling) and overnight with 10 μL of washed Protein G Sepharose 4B beads (Cat# 101242, Invitrogen). The beads were washed with coIP buffer and resuspended in 2X Laemmli buffer. Samples were fractionated on SDS–PAGE gels and analyzed using Western blotting.

### Fluorescence live cell imaging

Cells were fixed with 4% paraformaldehyde in PBS for 8 min at room temperature, washed three times with 1X PBS and then blocked with 10% serum and 0.3% Triton X-100 in PBS for 1 h at room temperature. Cells were again washed three times with 1X PBS and incubated a 1:200 dilution of rabbit polyclonal antibody against LC3B (Cat^#^ ab63817, Abcam) in PBS containing 0.1% Triton X-100 and 5% horse serum and overnight at 4 °C. After washing, the cells were incubated with fluorescein (FITC)-conjugated AffiniPure goat anti-mouse IgG (H + L) (Cat# SA00003-1, Proteintech) for 2 h at room temperature. Samples were mounted with Immu-mount (Thermo Scientific).

Cells were stained with 50 nM MitoTracker® Red CMXRos (Cat# 9082S, Cell Signaling Technology) for 30 min at 37 °C, washed with 1X PBS, and then used to detect the colocalization of LC3 and MitoTracker.

### Statistical analysis

Statistical analyses were conducted using GraphPad Prism 5 software. All data were tested for a normal distribution of variables. All normally distributed data are displayed as the means ± standard deviations (SD), unless indicated otherwise. Measurements between two groups were compared with an unpaired Student’s t test with Welch’s correction. Three or more groups were analyzed using one-way ANOVA followed by the Newman–Keuls test. Values of n for each experiment are reported in the figures and figure legends. P < 0.05 was considered significant. Statistical parameters for each experiment are provided within the corresponding figure legends.

## Results

### Bone marrow-specific deficiency of AIBP aggravates atherosclerosis in LDLR^−/−^ mice

**Fig. 1:**
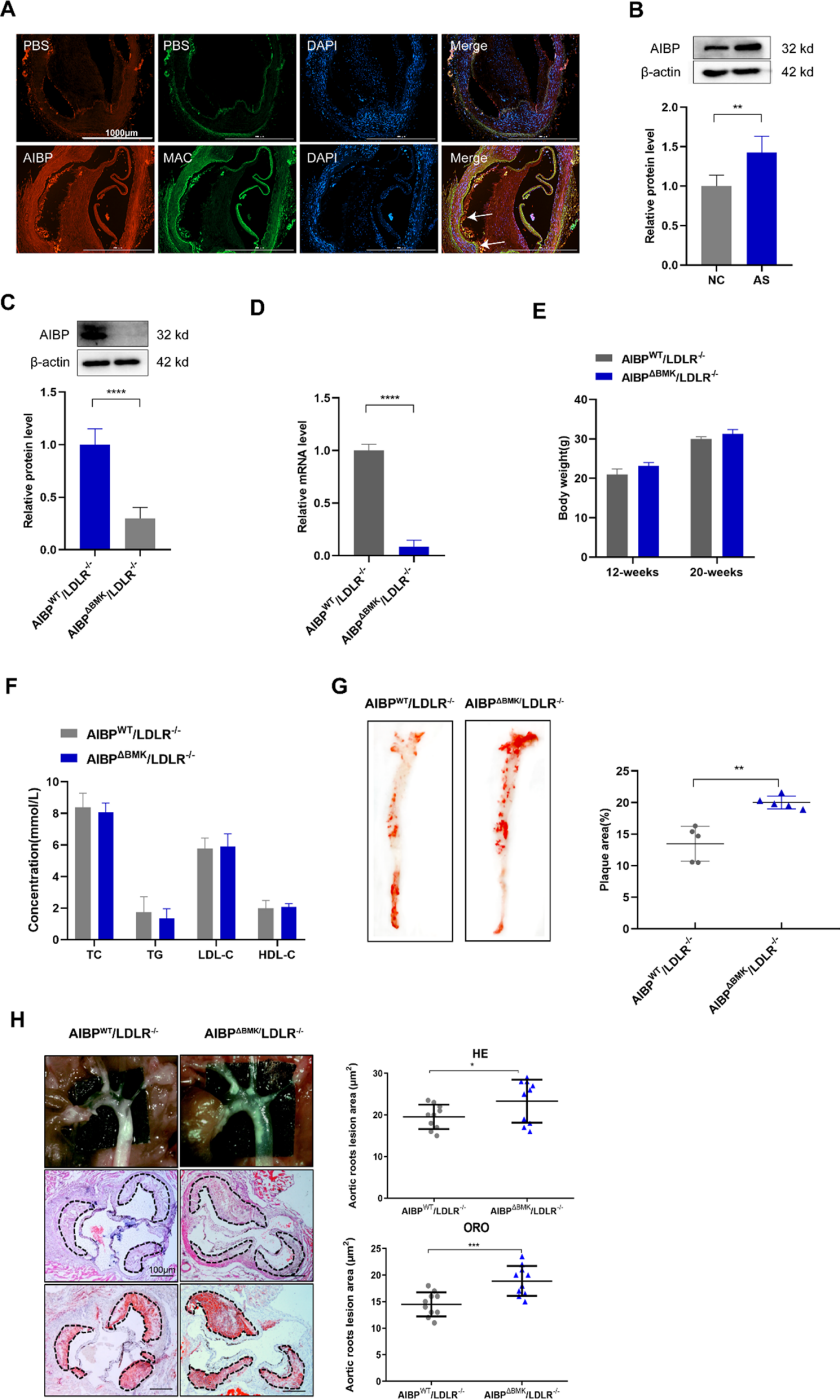
Bone marrow-specific deficiency of AIBP aggravates atherosclerosis in LDLR^−/−^ mice. **A** Representative images showing Mac3 and AIBP staining in human samples obtained from coronary artery plaques (n = 3). Scale bar = 1000 μm. **B** Western blot analysis of the AIBP protein level in mouse atherosclerotic plaques. β-Actin bands indicate the loading control (n = 5). **C** Western blot analysis of AIBP levels in BMDMs from AIBP^WT^/LDLR^−/−^ and AIBP^∆BMK^/LDLR^−/−^ mice. β-Actin bands indicate the loading control (n = 5). **D** qPCR analysis of the AIBP mRNA levels in BMDMs from AIBP^WT^/LDLR^−/−^ and AIBP^∆BMK^/LDLR^−/−^ mice. **E** Body weights of AIBP^WT^/LDLR^−/−^ and AIBP^∆BMK^/LDLR^−/−^ mice fed a HFD for 8 weeks (n = 5). **F** Comparison of the composition of serum lipids between AIBP^WT^/LDLR^−/−^ and AIBP^∆BMK^/LDLR^−/−^ mice fed a HFD for 8 weeks. TC (total serum cholesterol), TG (triglyceride), LDL (low-density lipoprotein) and HDL (high-density lipoprotein) contents were assayed (n = 5). **G** Oil Red O staining of aortic trees from AIBP^WT^/LDLR^−/−^ and AIBP^∆BMK^/LDLR^−/−^ mice. **H** Cross-sections of aortic roots from mice stained with hematoxylin and eosin (H&E) and Oil Red O (n = 10). Scale bar = 100 μm. Data are presented as the means ± SD. *p < 0.05, **p < 0.01, and ***p < 0.001. Unpaired two-tailed Student’s t test

First, we double-stained human atherosclerotic lesions with antibodies against AIBP and macrophage (Mac), smooth muscle cell (α-SMA) and endothelial cell (CD31) markers. We observed the colocalization of AIBP and macrophages (Fig. 1A), while smooth muscle cells and endothelial cells were not colocalized with AIBP (Additional file 1: Fig. S1A), indicating that intracellular AIBP may regulate macrophage function and the progression of atherosclerosis in addition to functioning as a secretory protein. We generated LDLR^−/−^ recipient mice transplanted with AIBP^−/−^ or their wild-type littermate donor bone marrow to study the effects of intracellular AIBP on atherosclerosis in vivo. As expected, the expression of AIBP in BMDMs from AIBP^ΔBMK^/LDLR^−/−^ mice was dramatically decreased compared with that in AIBP^WT^/LDLR^−/−^ mice (Fig. 1C, D), indicating that the AIBP^ΔBMK^/LDLR^−/−^ model was successfully established.

AIBP^WT^/LDLR^−/−^ and AIBP^∆BMK^/LDLR^−/−^ mice were fed a high-fat diet (HFD) for 8 weeks beginning at 12 weeks of age to evaluate the role of macrophage AIBP in the development of atherogenesis. As indicated in Fig. 1E, no remarkable changes in body mass index were observed between AIBP^WT^/LDLR^−/−^ and AIBP^∆BMK^/LDLR^−/−^ mice. Additionally, no statistically significant changes in total plasma cholesterol, LDL cholesterol, triglyceride, or HDL cholesterol levels were detected in mice fed the HFD for 8 weeks (Fig. 1F). Oil Red O staining showed significant increases in the number and size of lipid-laden plaque areas in the entire aorta of AIBP^ΔBMK^/LDLR^−/−^ mice compared to AIBP^WT^/LDLR^−/−^ mice (Fig. 1G). Consistently, histological analyses and Oil Red O staining of the tricuspid valve showed an increased atherosclerotic lesion area and lipid accumulation in AIBP^ΔBMK^/LDLR^−/−^ mice compared to AIBP^WT^/LDLR^−/−^ mice (Fig. 1H). Collectively, these results suggest that intracellular AIBP may play an antiatherosclerotic role.

### AIBP^ΔBMK^ promotes macrophage infiltration and proinflammatory polarization in LDLR^−/−^ mice

**Fig. 2:**
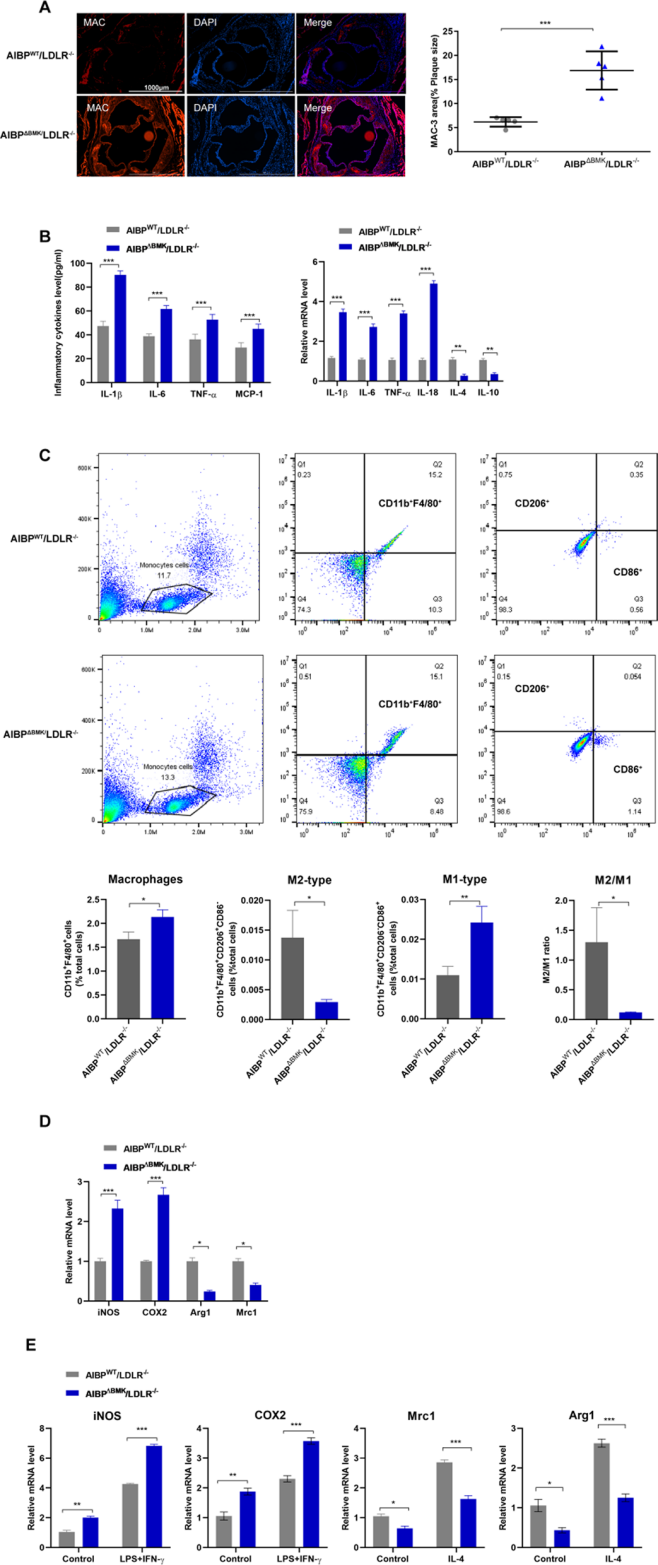
AIBP^ΔBMK^ promotes macrophage infiltration and proinflammatory polarization in LDLR^−/−^ mice. **A** Representative images of Mac3 staining in aortic roots isolated from AIBP^ΔBMK^/LDLR^−/−^ and AIBP^WT^/LDLR^−/−^ mice fed a high-fat diet (n = 3). Scale bar = 100 μm. **B** ELISA and qPCR were used to detect the protein and mRNA levels of inflammatory cytokines in the aorta (n = 5). **C** Flow cytometry analysis of macrophage infiltration and numbers of M1/M2-type macrophages in the blood of AIBP^ΔBM^K/LDLR^−/−^ and AIBP^WT^/LDLR^−/−^ mice after 8 weeks of HFD consumption. n = 4–5 mice per group. **D** qPCR analysis of the mRNA levels of macrophage phenotype markers in aortas from AIBP^ΔBMK^/LDLR^−/−^ and AIBP^WT/^LDLR^−/−^ mice (n = 5). **E** qPCR analysis of the mRNA levels of M1 and M2 markers in MPMs from AIBP^ΔBMK^/LDLR^−/−^ and AIBP^WT^/LDLR^−/−^ mice after 8 weeks of HFD consumption that were stimulated with LPS + IFN-γ or IL-4 (n = 5). Data are presented as the means ± SD. *P < 0.05, **P < 0.01, and ***P < 0.001; one-way ANOVA followed by the Newman–Keuls test

We observed the number of macrophages in AIBP^ΔBMK^/LDLR^−/−^ mice to determine the exact role of intracellular AIBP in atherosclerosis. Fluorescence staining revealed increased macrophage infiltration (Mac3 +) in the plaques of AIBP^ΔBMK^/LDLR^−/−^ mice compared to AIBP^WT^/LDLR^−/−^ mice (Fig. 2A). Consistent with this finding, the levels of proinflammatory mediators (IL-1β, IL-6, TNF-α, and IL-18) were increased, whereas the levels of anti-inflammatory mediators (IL-4 and IL-10) were decreased in the aortas and plasma of AIBP^ΔBMK^/LDLR^−/−^ mice (Fig. 2B). The macrophage microenvironment mainly depends on the composition of inflammatory factors, changes in which obviously switch the macrophage phenotype [32]. We therefore examined macrophage infiltration and numbers of M1/M2-type macrophages in the blood of AIBP^ΔBMK^/LDLR^−/−^ and AIBP^WT^/LDLR^−/−^ mice after 8 weeks of HFD consumption. As shown in Fig. 2C, markedly higher numbers of CD11b^+^F4/80^+^ macrophages were detected in AIBP^ΔBMK^/LDLR^−/−^ mice than in AIBP^WT^/LDLR^−/−^ mice. Consistent with this result, a substantial increase in the proportion of M1 macrophages (CD11b^+^F4/80^+^CD206^−^CD86^+^) and a significant reduction in the proportion of M2 macrophages (CD11b^+^F4/80^+^CD206^+^CD86^−^) were observed in AIBP^ΔBMK^/LDLR^−/−^ mice compared to AIBP^WT^/LDLR^−/−^ mice. The M2/M1 ratio was significantly decreased, indicating a shift in polarization toward the M1 type. In addition, the mRNA levels of macrophage phenotype markers in aortas from AIBP^ΔBMK^/LDLR^−/−^ and AIBP^WT^/LDLR^−/−^ mice were analyzed using qPCR. The results showed a tendency toward increased levels of M1 markers (iNOS and COX2); however, the levels of M2 markers (Arg1 and Mrc1) were decreased in AIBP^ΔBMK^/LDLR^−/−^ mice (Fig. 2D).

We isolated mouse peritoneal macrophages (MPMs) from AIBP^ΔBMK^/LDLR^−/−^ and AIBP^WT^/LDLR^−/−^ mice after 8 weeks of HFD consumption and stimulated them with LPS + IFNγ or IL-4 to further determine the function of AIBP in regulating the macrophage phenotype. Interestingly, increased mRNA levels of M1 marker genes but decreased levels of M2 marker genes were confirmed in LPS + IFNγ- or IL-4-treated MPMs from AIBP^ΔBMK^/LDLR^−/−^ mice compared with those from AIBP^WT^/LDLR^−/−^ mice (Fig. 2E). Based on these data, intracellular AIBP may be involved in the progression of inflammation in vivo by regulating macrophage polarization.

### Intracellular AIBP is located in the mitochondria of macrophages and inhibits inflammation

**Fig. 3:**
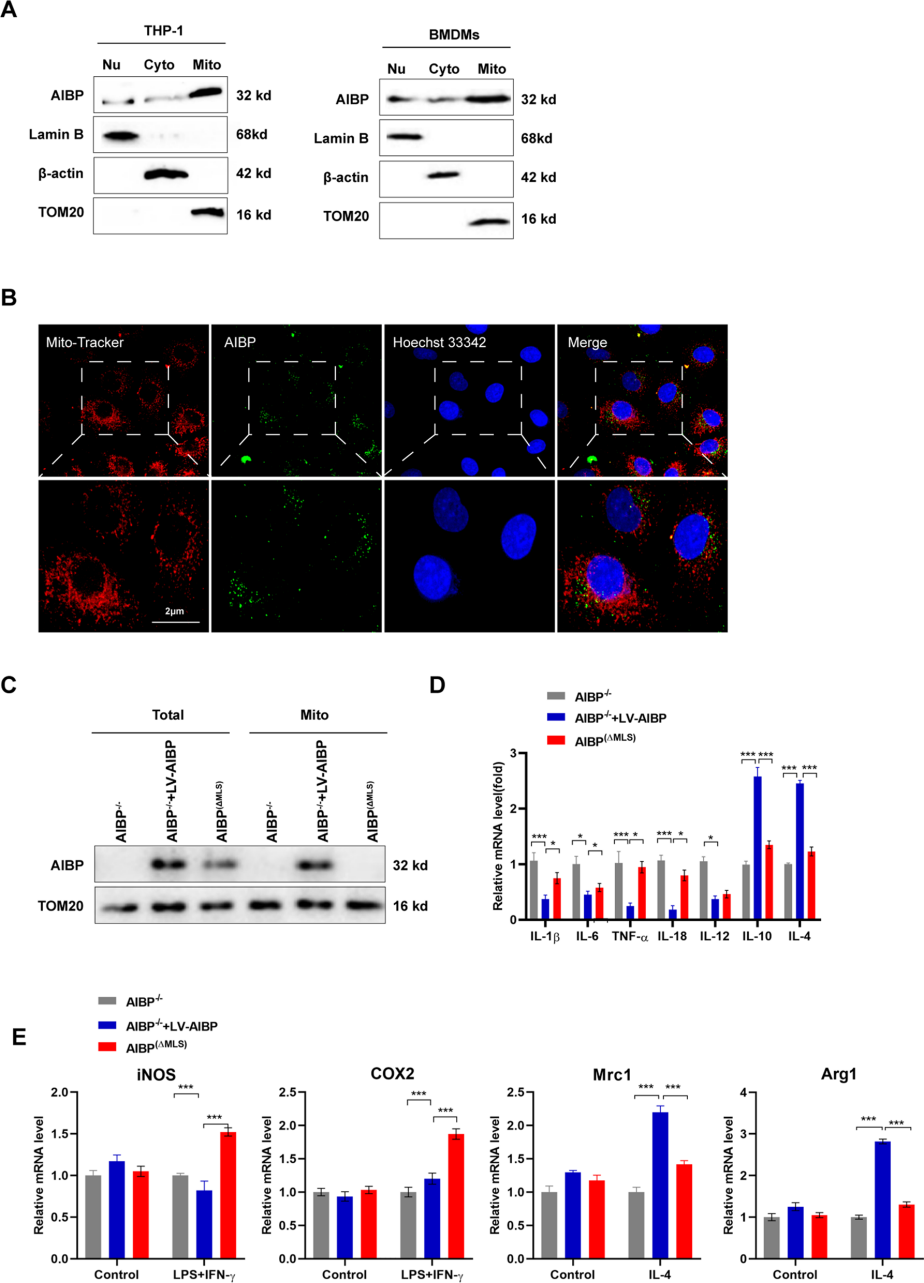
Intracellular AIBP is located in the mitochondria of macrophages and inhibits inflammation. **A** Western blot was used to determine AIBP levels in the nuclei and mitochondria of THP-1-derived macrophages and BMDMs (n = 3). **B** Live cell imaging of BMDMs using a confocal microscope. Mitochondria were stained with MitoTracker Green. Scale bar = 2 μm. **C** Western blot analysis of AIBP levels in isolated mitochondria and BMDMs (n = 3). **D** qPCR analysis of the mRNA levels of inflammatory cytokines in BMDMs. **E** qPCR analysis of the mRNA levels of M1 and M2 marker genes in infected BMDMs treated with LPS + IFN-γ or IL-4 (n = 5). Data are presented as the means ± SD. *P < 0.05, **P < 0.01, and ***P < 0.001. One-way ANOVA followed by the Newman–Keuls test

AIBP has also been described as a mitochondrial protein because it contains an N-terminal mitochondrial location sequence (MLS) [16]. Using the methods described by Marbaix et al. [16], mutation of the first methionine of the AIBP mitochondrial sequence to isoleucine suppressed mitochondrial labeling; as a result, the AIBP protein was only localized in the cytoplasm and nucleus. Consistent with this finding, we observed abundant AIBP protein expression in the mitochondrial fractions of human leukemic cell line (THP-1)-derived macrophages and BMDMs (Fig. 3A). Live cell imaging experiments showed obvious colocalization of AIBP and mitochondrial markers (Fig. 3B). We confirmed whether mitochondrial AIBP participated in regulating macrophage functions by isolating BMDMs from AIBP^−/−^ mice and transfecting them with a virus designed to overexpress the full-length AIBP sequence and restore AIBP expression (AIBP^−/−^ + LV-AIBP group), and then transfected an AIBP mutated in the N-terminal mitochondrial localization sequence (AIBP^−/−^ + LV-AIBP^(ΔMLS)^ group, abbreviated as the AIBP^(ΔMLS)^ group) to construct a mitochondrial AIBP-deficient cell model. In the AIBP^−/−^ + LV-AIBP group, AIBP was detected in total proteins and the mitochondrial fraction, while AIBP^(ΔMLS)^ was not expressed in mitochondria, indicating that the mitochondrial AIBP deficiency model was successfully established (Fig. 3C). We next detected decreased expression levels of the proinflammatory factors IL-1β, IL-6, IL-12, IL-18 and TNF-α, and the expression levels of the anti-inflammatory factors IL-4 and IL-10 were increased in the AIBP^−/−^ + LV-AIBP group compared to the AIBP^(ΔMLS)^ group (Fig. 3D). Furthermore, BMDMs were pretreated with LPS + IFNγ or IL-4 to induce M1 or M2 macrophage polarization. The AIBP^(ΔMLS)^ group displayed higher M1 marker and lower M2 marker expression than the AIBP^−/−^ + LV-AIBP group (Fig. 3E). These results prove the important function of intracellular AIBP in suppressing the inflammatory response by maintaining the phenotype of macrophages, at least part of which is maintained by mitochondrial AIBP.

### Mitochondrial AIBP maintains mitochondrial OXPHOS

**Fig. 4:**
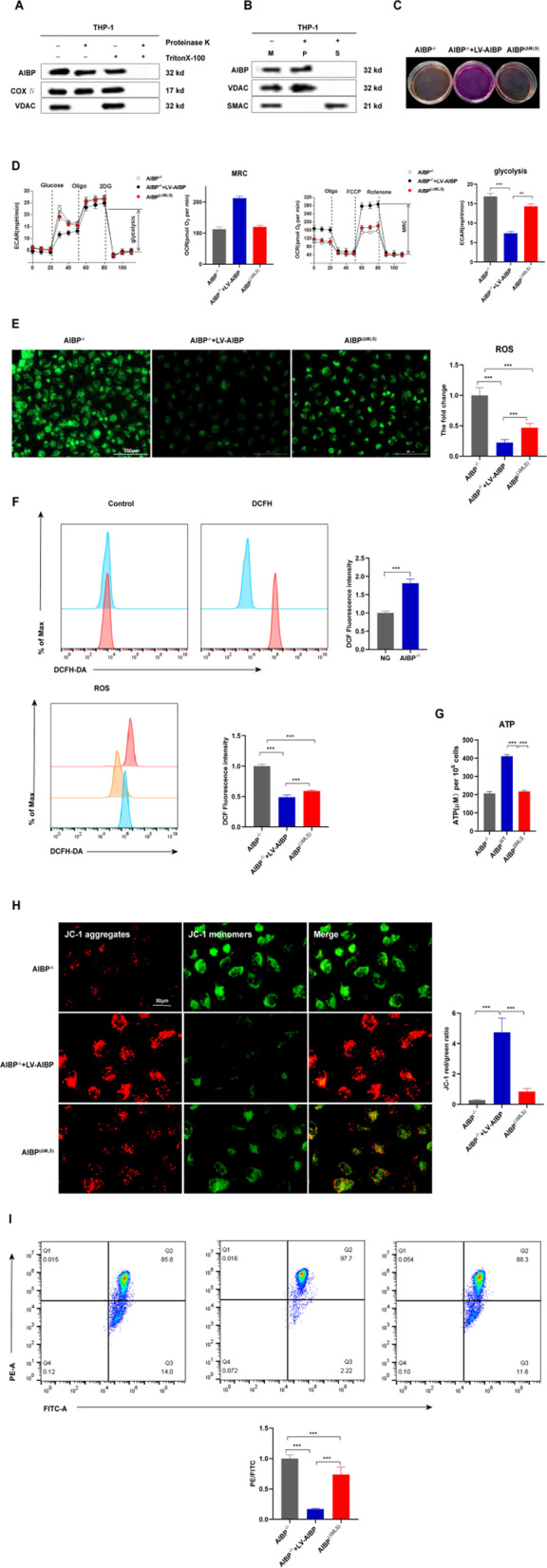
Mitochondrial AIBP maintains mitochondrial OXPHOS. **A**–**B** Alkaline extraction was performed using Na_2_CO_3_ to treat the mitochondrial fraction isolated from THP-1 cells. Both the soluble protein fraction (S) and integral membrane protein fraction (P) of AIBP were used for Western blot analysis. (M) Untreated control mitochondrial sample (n = 5). **C** Phenol red-containing cell culture media from AIBP^−/−^, AIBP^−/−^ + LV-AIBP and AIBP^(ΔMLS)^ BMDMs (n = 5). **D** BMDMs were seeded in Seahorse plates and incubated for 24 h. During the extracellular flux analysis, cells were sequentially treated with oligomycin (OM), carbonyl cyanide-4-(trifluoromethoxy) phenylhydrazone (FCCP), and rotenone (ROT) plus antimycin A (AA) to assess OXPHOS parameters related to the OCR levels or with glucose, oligomycin (OM), and 2-deoxyglucose (2-DG) to determine glycolysis parameters related to the ECAR levels. **E** Fluorescence images of BMDMs stained with 10 μM DCF-DA. Intracellular reactive oxygen species (ROS) generation was identified by measuring the DCF-DA intensity under a fluorescence microscope. Scale bar: 200 μm. **F** Flow cytometry analysis to detect ROS production. (**G**) ATP levels in AIBP^−/−^, AIBP^−/−^ + LV-AIBP and AIBP^(ΔMLS)^ BMDMs. **H** Cells were stained with JC-1. In nondamaged cells, JC-1 forms red-emitting aggregates in the mitochondrial matrix. A loss of red fluorescence and an increase in cytoplasmic green-emitting monomers signal the disruption of the mitochondrial transmembrane potential (ΔΨm). Scale bar: 50 μm. **I** ΔMφ was measured using flow cytometry after staining BMDMs with tetramethylrhodamine methylester (TMRM). Data are presented as the means ± SD. *P < 0.05, **P < 0.01, and ***P < 0.001. One-way ANOVA followed by the Newman–Keuls test

The subcellular localization of a protein is closely related to and indicates its function. Although mitochondrial AIBP is involved in regulating macrophage functions, its subcellular localization is not well defined. We found that AIBP is located in mitochondria in THP-1 cells (Fig. 4A). Next, we isolated mitochondrial proteins to determine the specific site of AIBP localization in mitochondria. Alkaline lysis showed that voltage-dependent anion channel (VDAC) and AIBP were primarily recovered in the pellet following extraction (Fig. 4B), further indicating that AIBP is located in the mitochondrial inner membrane in THP-1 cells. According to a previous study, AIBP is related to the metabolism of NADPH derivatives and is closely associated with OXPHOS [33]. The function seems to depend on the location of AIBP in the mitochondrial inner membrane; however, the exact effect has not been clarified. Therefore, we further explored whether mitochondrial AIBP regulated mitochondrial metabolism. First, we observed that AIBP^(ΔMLS)^ BMDMs produced more acidic molecules than AIBP^−/−^ + LV-AIBP BMDMs (Fig. 4C). Thus, mitochondrial metabolism may have changed due to the deficiency of mitochondrial AIBP. Next, a series of mitochondrial metabolism-related indicators, such as the extracellular acidification rate (ECAR), which indicates aerobic glycolysis, and the oxygen consumption rate (OCR), which indicates OXPHOS, were examined. The OCR in AIBP^(ΔMLS)^ BMDMs displaying the maximum respiratory capacity (MRC) was notably decreased. In addition, a noticeable shift in glycolysis was detected in AIBP^(ΔMLS)^ BMDMs and AIBP^−/−^ BMDMs (Fig. 4D). Furthermore, OXPHOS dysfunction is known to lead to reactive oxygen species (ROS) production and changes in the mitochondrial membrane potential (ΔMφ) and ATP levels. As shown in Fig. 4E–G, increased mitochondrial ROS (mtROS) and reduced ΔMφ and ATP levels were identified in AIBP^(ΔMLS)^ BMDMs compared with AIBP^−/−^ + LV-AIBP BMDMs. Based on these results, mitochondrial AIBP plays an essential role in mitochondrial metabolism.

### Mitochondrial AIBP maintains the macrophage phenotype by altering mitophagy through mitochondrial OXPHOS

**Fig. 5:**
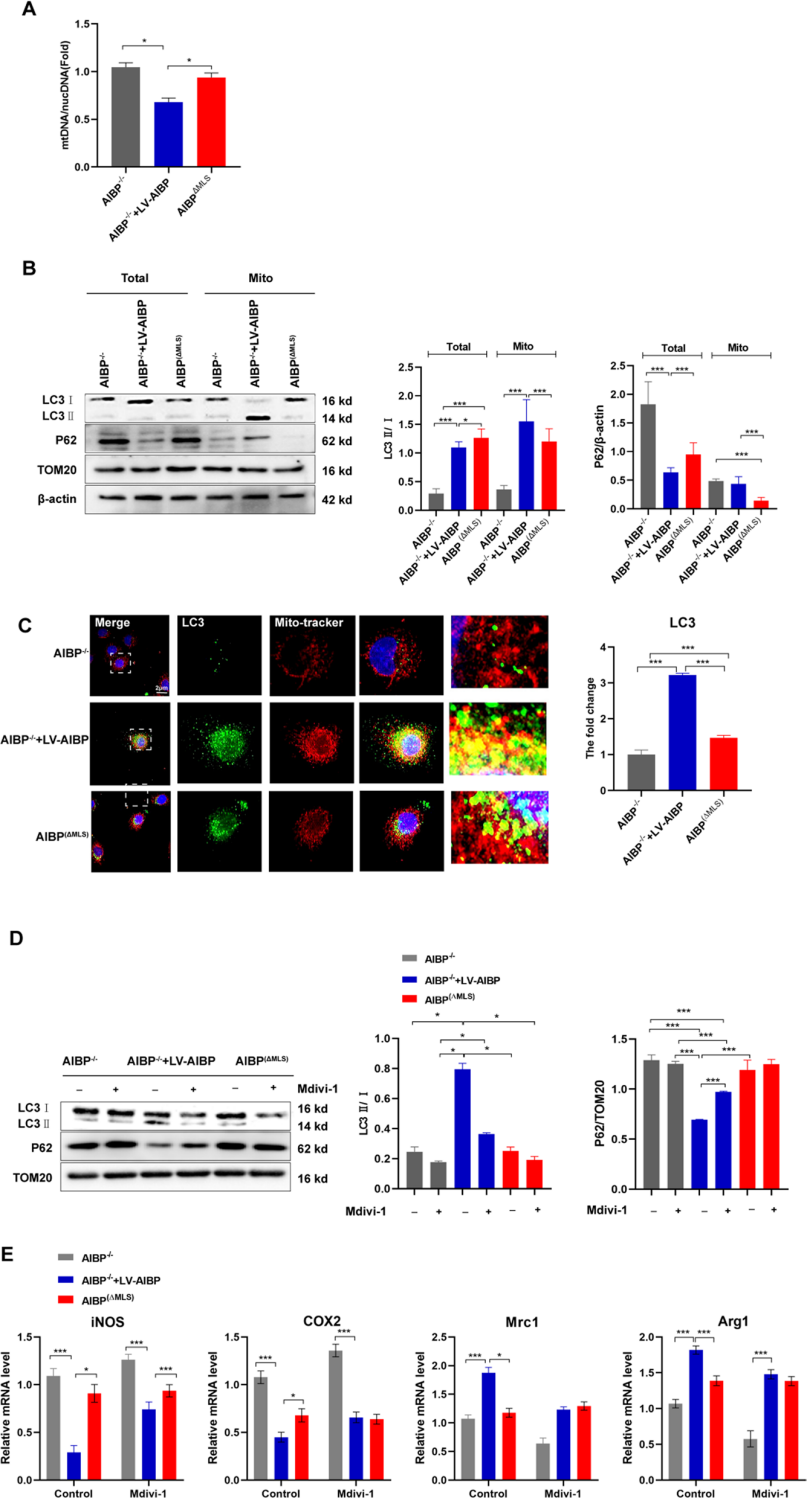
Mitochondrial AIBP maintains the macrophage phenotype by altering mitophagy through mitochondrial OXPHOS. **A** The ratio of mitochondrial DNA (mtDNA) to nuclear DNA (nucDNA) in AIBP^−/−^, AIBP^WT^ or AIBP ^(ΔMLS)^ BMDMs was evaluated using qPCR (n = 5). **B** Western blot analysis of LC3b, p62 and TOM20 expression in BMDMs. **C** Live cell imaging of BMDMs using a confocal microscope. Mitochondria were stained with MitoTracker Green. Scale bar = 2 μm. **D** Western blot analysis of the expression of LC3b, p62 and TOM20 in BMDMs after treatment with mitochondrial division inhibitor 1 (mdivi-1). **E** qPCR analysis of the mRNA levels of M1 marker genes in BMDMs stimulated with LPS + IFN-γ after the Mdivi1 pretreatment and M2 marker genes in BMDMs stimulated with IL-4 after the Mdivi1 pretreatment (n = 5). Data are presented as the means ± SD. *P < 0.05, **P < 0.01, and ***P < 0.001. One-way ANOVA followed by the Newman–Keuls test

A loss of the ΔMφ in mitochondria is closely related to mitophagy, which ordinarily occurs in response to mitochondrial dysfunction [32]. Therefore, we examined the mitochondrial DNA content in BMDMs. With the decrease in ΔMφ, the DNA content of the AIBP^(ΔMLS)^ group increased (Fig. 5A), suggesting that mitophagy may be impaired. Based on these data, we hypothesized that mitochondrial AIBP deletion impaired mitophagy, damaging mitochondrial quality control. We examined levels of the mitophagy markers (LC3bII and p62) and a mitochondrial marker (TOM20) to assess this hypothesis. The results showed decreased LC3bII levels in the AIBP^(ΔMLS)^ group compared to the AIBP^−/−^ + LV-AIBP group (Fig. 5B). Remarkably, live cell imaging showed little overlap between MitoTracker and LC3bII in AIBP^(ΔMLS)^ (Fig. 5C). In addition, we used mitochondrial division inhibitor 1 (mdivi-1) to further show that the lack of mitochondrial AIBP in macrophages impaired mitophagy (Fig. 5D).

Next, we used mdivi-1 to observe its effect on macrophage polarization. Mdivi-1 partially blocked the downregulation of the M1 markers COX2 and iNOS, while it significantly increased the mRNA levels of Arg1 and Mrc1 in AIBP^(ΔMLS)^ BMDMs (Fig. 5E). Consistent with these results, mitochondrial AIBP regulates macrophage polarization via mitochondrial autophagy.

### AIBP deficiency inhibits mitophagy via PINK1 cleavage

**Fig. 6:**
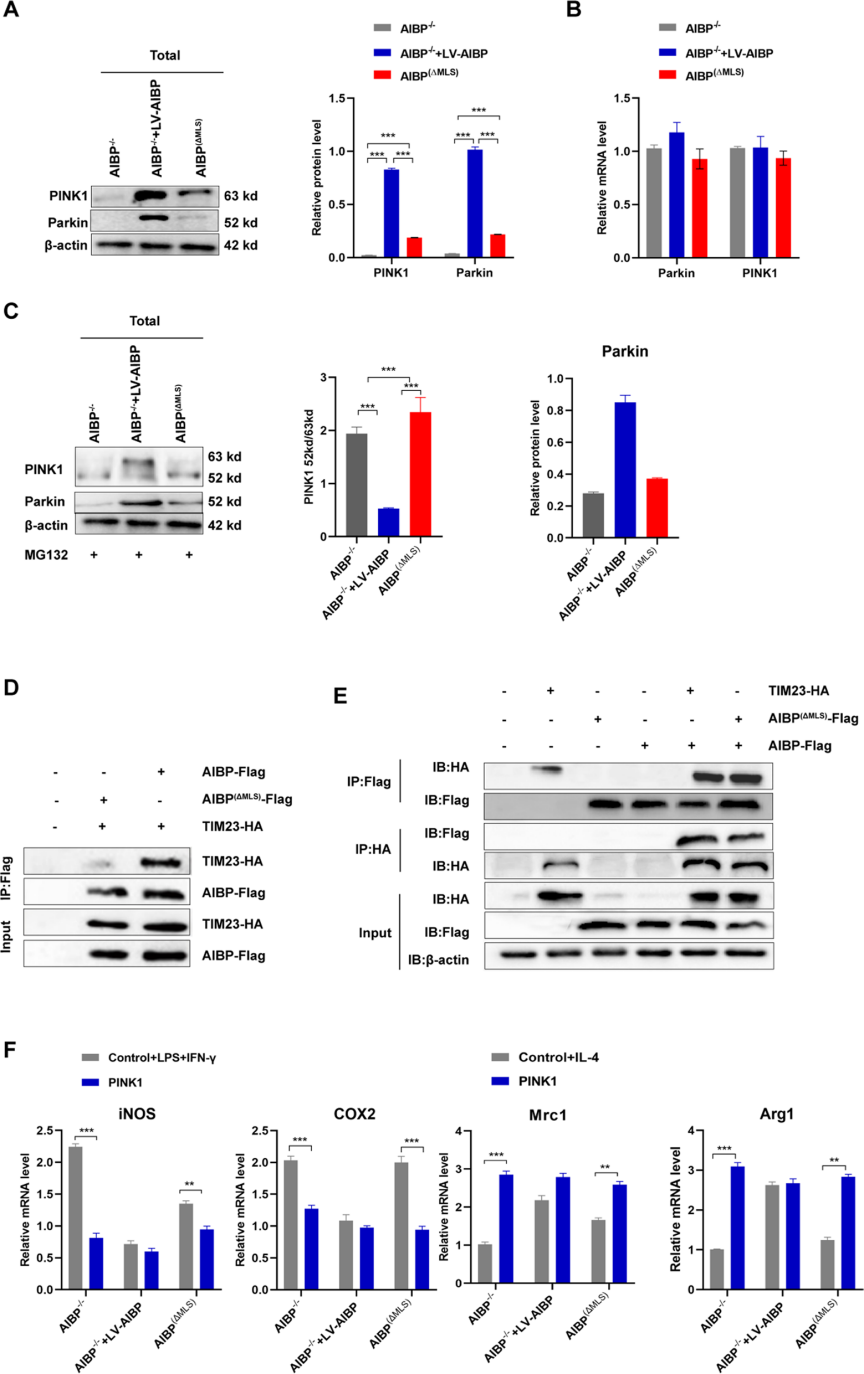
AIBP deficiency inhibits mitophagy via PINK1 cleavage. **A** Western blot analysis of the levels of Parkin and PINK1 in the total lysate of BMDMs (n = 5). **B** qPCR analysis of the Parkin and PINK1 mRNA levels in BMDMs (n = 5). **C** Western blot analysis of PINK1 and Parkin levels in the total lysate of BMDMs (n = 5). **D**–**E** The interaction of AIBP with TIM23 in 293 T cells was assessed by performing coimmunoprecipitation and Western blot assays. **F** qPCR was performed to detect the mRNA levels of M1 markers and M2 markers in MPMs extracted from AIBP^−/−^, AIBP^WT^ or AIBP^(ΔMLS)^ mice in the LV-control (control) group and LV-PINK1 (PINK1) group that were separately stimulated with LPS + IFN-γ and IL-4 (n = 5). Data are presented as the means ± SD. *P < 0.05, **P < 0.01, and ***P < 0.001. One-way ANOVA followed by the Newman–Keuls test

Activation of PINK1/Parkin signaling contributes to the induction of mitophagy [34].However, when imported to the mitochondria, full-length (63 kDa) PINK1 is cleaved, generating a 52 kDa PINK1 fragment that undergoes proteasomal degradation [35]. Our results showed a reduction in PINK1 and Parkin protein expression, while their mRNA levels were not obviously altered in AIBP^(ΔMLS)^ BMDMs compared to AIBP^WT^ BMDMs (Fig. 6A, B), suggesting that the change in the PINK1 protein content may be due to its degradation. In addition, the proteasome inhibitor MG132 markedly increased levels of the 52 kDa PINK1 fragment in AIBP^(ΔMLS)^ BMDMs, further indicating that mitochondrial AIBP may be involved in PINK1 cleavage in mitochondria (Fig. 6C). Then, we explored the coupling of AIBP to the mitochondrial inner membrane transporter (TIM) TIM23, and found that AIBP was located in the mitochondrial inner mitochondrial membrane (IMM) (Fig. 6D). Finally, we overexpressed PINK1 and observed its effect on the macrophage phenotypic polarization. The evidence shows that overexpression of PINK1 reversed the polarization of macrophages to the M1 phenotype (Fig. 6E). Taken together, our results indicate that mitochondria-localized AIBP may regulate mitochondrial autophagy by altering the degradation of PINK1.

## Discussion

Most previous studies have focused on the role of AIBP as a secreted protein. In zebrafish and mouse models, AIBP deficiency leads to accelerated angiogenesis, increased inflammation and increased atherosclerosis [15].An external injection of AIBP reduces aortic inflammation and atherosclerotic plaque formation [36]. In vitro experiments have revealed that AIBP promotes cholesterol efflux from endothelial cells and macrophages mediated by ATP-binding cassette transporter A1 (ABCA1) through its interaction with apoA-I [9, 10]. Based on these results, secreted AIBP regulates the immune/inflammatory response by regulating lipid transport and lipid raft-related receptor activity. Therefore, this information provides a future research direction for methods to control AIBP secretion in the body and to target and regulate the occurrence of diseases by administering exogenous AIBP in vivo.

The human AIBP gene is associated with familial combined hyperlipidemia and inflammation [37]. AIBP expression has not been detected in serum from healthy donors, whereas it has been detected in serum from patients with septic syndromes [8]. Consistent with this observation, Choi et al. [38] and our group found that AIBP was expressed at high levels in macrophages from human atherosclerotic plaques. This peculiar localization implies the role of macrophage AIBP in cardiovascular functions. Schneider et al. [36] found that AIBP^−/−^/LDLR^−/−^ mice fed a high-cholesterol, high-fat diet exhibited exacerbated weight gain, liver steatosis, glucose intolerance, hypercholesterolemia, hypertriglyceridemia, and larger atherosclerotic lesions compared to LDLR^−/−^ mice. In addition, a report examining systemic metabolites in plasma samples from AIBP knockout mice indicated that AIBP may play an important role in regulating cellular metabolism [39]. In the present study, we used bone marrow transplantation (BMT) to transplant bone marrow from AIBP knockout mice or their wild-type littermate controls into LDL receptor knockout mice to construct AIBP^WT^/LDLR^−/−^ and AIBP^ΔBMK^/LDLR^−/−^ mouse models. After 8 weeks of HFD feeding, AIBP^ΔBMK^/LDLR^−/−^ showed severe atherosclerotic lesions, inflammatory cell infiltration and polarization of macrophages to the proinflammatory phenotype compared with AIBP^WT^/LDLR^−/−^ mice, indicating that intracellular AIBP may exert anantiatherosclerotic effect. A previous study has reported that BMT normally affects the development of aortic atherosclerosis in mice [40], but the application of this modality in atherosclerosis is mixed and warrants further investigation [41, 42]. In the present study, we used mouse littermates with different genotypes and simultaneously performed BMT transplantation controls. Although the genetic background of the recipient mice and the donor mice is different, the AIBP^ΔBMK^/LDLR^−/−^ mice in the experimental group have the same background as the AIBP^WT^/LDLR^−/−^ mice in the control group. Although the genetic background of the recipient mice and the donor mice is different, the AIBP^ΔBMK^/LDLR^−/−^ mice in the experimental group have the same background as the AIBP^WT^/LDLR^−/−^ mice in the control group. Thus, the influence of intracellular AIBP in atherosclerosis is strongly supported. However, further validation with macrophage-specific knockout of AIBP mice is required.

In atherosclerotic tissue, macrophages migrate and accumulate in response to inflammatory signals [43]. Changes in microenvironmental stimuli lead to rapid macrophage polarization into the M1 or M2 phenotype [44]. M1 macrophages are considered more proinflammatory and form the first line of defense against pathogens [44]. In comparison, M2 macrophages are considered anti-inflammatory and activate tissue repair and fibrosis [44]. A finding in NAXE deficient individuals showed that AIBP may be involved in aerobic metabolism [18]. In fact, both total deficiency and mitochondrial-specific deficiency of AIBP in BMDMs caused prominent macrophage polarization. Several studies have suggested that secreted AIBP is associated with atheroprotective functions in macrophages [11, 13], although the role of intercellular AIBP in mitochondria was largely unclear. Here, we showed that AIBP is expressed in mitochondria and plays an important role in macrophage polarization during atherosclerosis. Moreover, we observed a pronounced change in the culture supernatant pH after mitochondrial AIBP deletion in BMDMs, suggesting that AIBP might be a crucial regulator of the glycolytic shift. Thus, AIBP may be a potential target for controlling mitochondrial metabolism.

The mitochondrion is a double membrane-bound organelle with a vital role in cell metabolism, apoptosis and polarization [45, 46]. An important metabolic program, OXPHOS, occurs in the mitochondrial inner membrane [47]. AIBP is an epimerase that is involved in NADH recycling [16], and various metabolic enzymatic systems and is imperative for OXPHOS [48]. NADHX has been recognized to participate in various metabolic pathways and is linked to mitochondrial dysfunction in a reported case study of patients carrying biallelic NAXD mutations [49]. When OXPHOS dysfunction is present, higher ROS and lower ΔMφ levels are observed [50]. Here, we have shown that mitochondrial AIBP deficiency causes mitochondrial damage by inducing a tremendous glycolysis shift in BMDMs. Mitochondrial metabolic reprogramming-dependent macrophage polarization from the M1 to M2 phenotype is affected by mitophagy in the inflammatory environment [51]. Under pathological conditions, the increase in proinflammatory cytokines leads to the accumulation of mROS, resulting in increased mitochondrial damage and causing inflammation and vascular damage [52, 53]. Thus, mROS levels act as a sensor for mitophagy, maintaining mitochondrial quality control [54].

Mitophagy is a vital process for the elimination of dysfunctional mitochondria [55, 56]. The results obtained from multiple cell and animal models indicate that the regulation of autophagy plays an important role in both early and advanced atherosclerosis [57]. Choi et al. [38] found that macrophage AIBP enhances mitochondrial autophagy and participates in mitochondrial quality control, protecting macrophages from death in the context of atherosclerosis. However, part of the content of our manuscript overlaps with the study by Choi et al. In fact, we have been committed to the studying the role of the AIBP protein in macrophage-mediated inflammation for many years [13, 17]. In this manuscript, we explored the relationship between mitochondrial AIBP and mitochondrial autophagy and macrophage polarization by constructing a mitochondrial AIBP deletion vector. At present, research on AIBP mainly focuses on the field of lipid transport and its role as a secreted protein, ignoring the special role of AIBP expressed in mitochondrian [9, 10, 58]. In the future, important goals will be to determine the functions of differently located AIBP and to more comprehensively elucidate the role of AIBP in mitochondria.

PINK1 is a serine/threonine kinase that is located on the outer mitochondrial membrane when mitochondrial function is abnormal and then recruits Parkin for Parkin-dependent target protein ubiquitination on the outer mitochondrial membrane [55]. Ubiquitous substrates are recognized by adaptor proteins, which mediate the recruitment of LC3bII, followed by mitophagy initiation [59]. Full-length (63 kDa) PINK1 is transported into mitochondria by the translocase of the outer mitochondrial membrane (TOM) and translocase of the inner mitochondrial membrane (TIM) and then rapidly cleaved, generating a 52 kDa PINK1 fragment that undergoes proteasomal degradation [35]. Our data showed that mitochondrial AIBP knockdown reduced the PINK1 protein level, while the mRNA level did not change, suggesting that accelerated PINK1 degradation may play a tangible role in the change in its protein level. Mitochondrial dysfunction impairs the TIM mediating PINK1 transport into mitochondria, which contributes to the prevention of PINK1 degradation and enables mitochondria to achieve mitochondrial quality control through mitophagy [59]. Here, our findings first revealed a potential location of AIBP in the inner mitochondrial membrane and the interaction of TIM with AIBP. Liu et al. [60] showed that mature 52 kDa PINK1 is the target of ubiquitination. However, in the study by Choi et al. [38], mitochondria-localized AIBP interacted with the E3 ubiquitin-protein ligases PARK2 (Parkin), MFN (mitofusin)1, and MFN2 via its N-terminal domain, but not BNIP3 (Bcl2/adenovirus E1B 19-kDa-interacting protein-3), and regulated the ubiquitination of MFN1 and MFN2, key components of mitophagy. Therefore, AIBP and the level of ubiquitination are closely related.

In conclusion, our findings revealed a novel role for mitochondrial AIBP in atherosclerosis by regulating macrophage polarization. Furthermore, AIBP deletion induced dysfunctional mitochondrial metabolism, which resulted in the occurrence of mitophagy via the classical PINK1/Parkin pathway. Given the protective effect of mitochondrial AIBP on atherosclerosis, increasing the AIBP level may be an effective therapeutic approach for the treatment of atherosclerosis.

## Conclusions

In summary, our findings reveal a new role for AIBP in atherosclerosis, and its deficiency in macrophages exacerbates atherosclerosis and inflammation. Increased inflammation in atherosclerosis may be caused by increased PINK1 cleavage in mitochondria and impaired mitophagy. Due to the protective effect of mitochondrial AIBP on atherosclerosis, this protein may be an effective target for the treatment of chronic inflammatory diseases characterized by macrophage dysfunction.

## Supplementary Information

**Additional file 1.** The smooth muscle cell marker α-SMA and the endothelial cell marker CD31 do not significantly colocalize with AIBP in human samples obtained from coronary artery plaques.

**Additional file 2: Table S1**: Sequences of the primers used for the different constructs.

**Additional file 3: Table S2.** The primers of qPCR.

## Data Availability

The data generated are included in the manuscript and supplementary data.

## Abbreviations

AIBP: Apolipoprotein A-I binding protein
apoA-I: Apolipoprotein A-I
ABCA1: ATP-binding cassette transporter A1
Arg1: Arginase1
As: Atherosclerosis
BMDMs: Bone marrow-derived macrophages
COX2: Cyclooxygenase 2
CVD: Cardiovascular disease
ECAR: Extracellular acidification rate
HDL: High-density lipoprotein
iNOS: Inducible nitric oxide synthase
MLS: Mitochondrial location sequence
MRC: Maximum respiratory capacity
mROS: Mitochondrial reactive oxygen species
MPMs: Mouse peritoneal macrophages
Mrc1: Mannose receptor 1
NADH: Nicotinamide adenine dinucleotide
NADHX: Nicotinamide adenine dinucleotide hydrate
NAD (P) HX: Nicotinamide adenine dinucleotide phosphate reduced form hydrate
OCR: Oxygen consumption rate
OXPHOS: Oxidative phosphorylation
PINK1: PTEN induced putative kinase 1
THP-1: Human leukemic cell line
TIM: The inner mitochondrial membrane
TLR4: Toll-like receptor 4
TOM: Translocator in the outer mitochondrial membrane
ΔMφ: Mitochondrial membrane potential
