# Supplementary material for: Mitochondrial apolipoprotein A-I binding protein alleviates atherosclerosis by regulating mitophagy and macrophage polarization
Source: Cell Commun Signal. 2022 May 7;20:60. doi: 10.1186/s12964-022-00858-8 (PMC9077873; doi:10.1186/s12964-022-00858-8)
Supplement: Supplementary file 2 — Additional file 1. The smooth muscle cell marker α-SMA and the endothelial cell marker CD31 do not significantly colocalize with AIBP in human samples obtained from coronary artery plaques. [file 12964_2022_858_MOESM2_ESM.docx]

# Supplementary Table 2


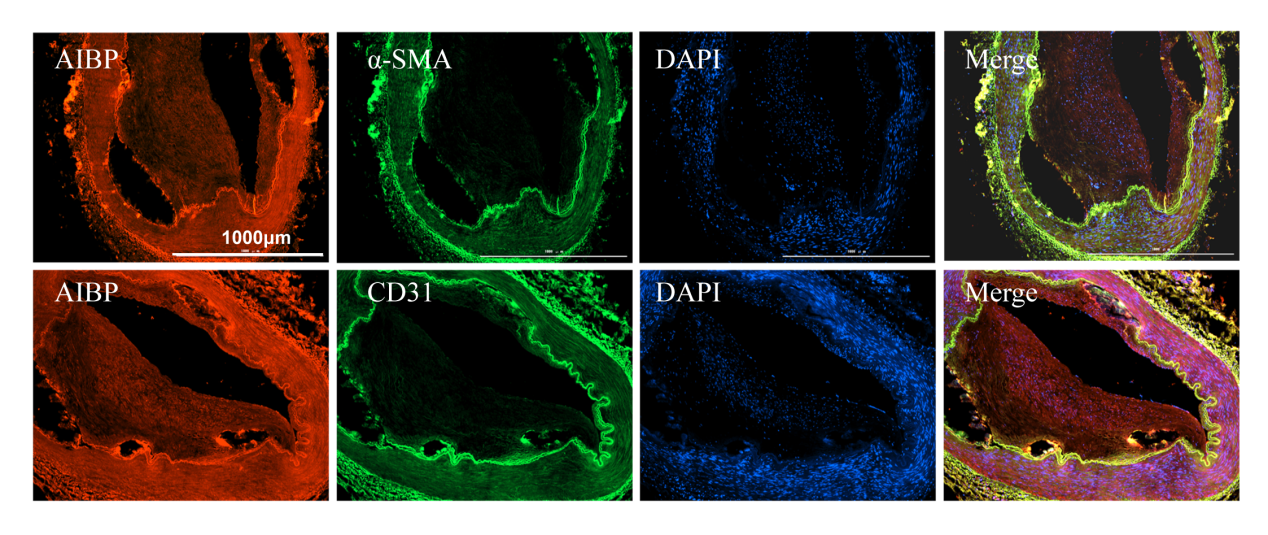


**Fig. 1 The smooth muscle cell marker α-SMA and the endothelial cell marker CD31 do not significantly colocalize with AIBP in human samples obtained from coronary artery plaques**

**(A)** Representative images showing α-SMA, CD31 and AIBP staining in human samples obtained from coronary artery plaques (n=3). Scale bar=1000 μm.
