# Supplementary material for: Mitochondrial apolipoprotein A-I binding protein alleviates atherosclerosis by regulating mitophagy and macrophage polarization
Source: Cell Commun Signal. 2022 May 7;20:60. doi: 10.1186/s12964-022-00858-8 (PMC9077873; doi:10.1186/s12964-022-00858-8)
Supplement: Supplementary file 3 — Additional file 2: Table S1: Sequences of the primers used for the different constructs. [file 12964_2022_858_MOESM3_ESM.docx]

# Supplementary Table 1

Sequences of the primers used for the different constructs

| Construct | Forward Primers | Reverse Primers |
| --- | --- | --- |
| LV-AIBP | GGGGAATTCGCCACCATGTCCGGGCTGCGGACGCTGCTGGGG | GGGGGATCCTTACTGTAGACGGTAGACACACTCT |
| LV-AIBP^ΔMLS^ | GCTACCGGACTCAGATCTATCTCCGGGCTGCGGACGCTG | CAGCGTCCGCAGCCCGGAGATAGATCTGAGTCCGGTAGC |
| LV-PINK1 | TCCGCTCGAGATGGCGGTGCGACAGGCGCT | ATCGGGATCCCCGCAGGGCTGCCCTCCATGAG |
