# Supplementary material for: Mitochondrial apolipoprotein A-I binding protein alleviates atherosclerosis by regulating mitophagy and macrophage polarization
Source: Cell Commun Signal. 2022 May 7;20:60. doi: 10.1186/s12964-022-00858-8 (PMC9077873; doi:10.1186/s12964-022-00858-8)
Supplement: Supplementary file 4 — Additional file 3: Table S2. The primers of qPCR. [file 12964_2022_858_MOESM4_ESM.docx]

# Supplementary Table 2

The primers of qPCR

| Genes | Forward Primers | Reverse Primers |
| --- | --- | --- |
| IL-1β | CGTGGACCTTCCAGGATGAG | CATCTCGGAGCCTGTAGTGC |
| IL-6 | CGGCCTTCCCTACTTCACAA | TTCTGCAAGTGCATCATCGT |
| TNFα | TGAGCACAGAAAGCATGATCC | GCCATTTGGGAACTTCTCATC |
| IL-4 | GAGACTCTTTCGGGCTTT | ATGCTCTTTAGGCTTTCCA |
| IL-18 | GAAGAAGCCATAGACAcCAAGAG | AGGcGAGAACAAGCACAGT |
| IL-12 | CTGGAACTACACAAGAACGAGAG | GGCACAGGGTCATCATCAAAG |
| IL-10 | GAAGAAGcCATAGACAcCAAGAG | AGGCGAGAACAAGCACAGT |
| iNOS | GCTTGCCCCTGGAAGTTTCT | CCTCACATACTGTGGACGGG |
| COX2 | CTGACCCCCAAGGCTCAAAT | TCCATCCTTGAAAAGGCGCA |
| Arg1 | CTTGCGAGACGTAGACCCTG | CTTCCTTCCCAGCAGGTAGC |
| Mrc1(CD206) | GTGGAGTGATGGAACCCCAG | CTGTCCGCCCAGTATCCATC |
| PINK1 | TTCTTCCGCCAGTCGGTAG | CTGCTTCTCCTCGATCAGCC |
| Parkin | TCTTCCAGTGTAACCACCGTC | GGCAGGGAGTAGCCAAGTT |
| β-actin | GGCACCACACCTTCTACAATG | GTGGTGGTGAAGCTGTAGCC |
| AIBP | CCGGAATTCCATGTCCAGGCTGCGGGCGCTGCTGGGCCTCG | CGGGGTACCTCACTGCAGACGATAGACACACTC |
